# miRGate: a curated database of human, mouse and rat miRNA–mRNA targets

**DOI:** 10.1093/database/bav035

**Published:** 2015-04-08

**Authors:** Eduardo Andrés-León, Daniel González Peña, Gonzalo Gómez-López, David G. Pisano

**Affiliations:** ^1^Bioinformatics Unit (UBio), Structural Biology and Biocomputing Programme, Spanish National Cancer Research Centre (CNIO), Madrid, Spain and ^2^High Technical School of Computer Engineering, University of Vigo, Ourense, Spain

## Abstract

MicroRNAs (miRNAs) are small non-coding elements involved in the post-transcriptional down-regulation of gene expression through base pairing with messenger RNAs (mRNAs). Through this mechanism, several miRNA–mRNA pairs have been described as critical in the regulation of multiple cellular processes, including early embryonic development and pathological conditions. Many of these pairs (such as miR-15 b/BCL2 in apoptosis or BART-6/BCL6 in diffuse large B-cell lymphomas) were experimentally discovered and/or computationally predicted. Available tools for target prediction are usually based on sequence matching, thermodynamics and conservation, among other approaches. Nevertheless, the main issue on miRNA–mRNA pair prediction is the little overlapping results among different prediction methods, or even with experimentally validated pairs lists, despite the fact that all rely on similar principles. To circumvent this problem, we have developed miRGate, a database containing novel computational predicted miRNA–mRNA pairs that are calculated using well-established algorithms. In addition, it includes an updated and complete dataset of sequences for both miRNA and mRNAs 3′-Untranslated region from human (including human viruses), mouse and rat, as well as experimentally validated data from four well-known databases. The underlying methodology of miRGate has been successfully applied to independent datasets providing predictions that were convincingly validated by functional assays. miRGate is an open resource available at http://mirgate.bioinfo.cnio.es. For programmatic access, we have provided a representational state transfer web service application programming interface that allows accessing the database at http://mirgate.bioinfo.cnio.es/API/

Database URL: http://mirgate.bioinfo.cnio.es

## Introduction

In the past few years, the functional role of non-coding RNAs have been associated to crucial cellular processes, such as gene regulation (1) and chromatin modification (2). This evidence has been supported by the Encyclopedia of DNA Elements project which revealed that most of our non-coding genome is actively transcribed and that a substantial percentage of the genome is active at the transcriptional level (3). Among non-coding RNAs, the microRNAs (miRNAs) family has become relevant by their important regulatory role. miRNAs are small non-coding elements of ∼22 nt involved in the post-transcriptional fine-tuning regulation of gene expression, either through messenger RNA (mRNA) degradation or by translation prevention (4, 5). Recently, other mechanisms such as elongation inhibition or ribosome drop-off (premature termination) have been described (5). miRNAs have also been associated with many other relevant functions: apoptosis, cell growth, cell proliferation and differentiation in prokaryotes and eukaryotes organisms (6, 7). Several independent studies have predicted that miRNAs regulate 20–30% of human genes, but some authors raise this estimate considerably to 92% (8, 9). Alterations of the expression patterns of multiple miRNAs have been associated to pathological conditions such as cancer (10, 11), neurodegenerative diseases (12) and heart diseases (13).

Basic miRNA mechanism of action relies on binding their seed sequence (an evolutionary-conserved region of 5–7 nt at the 5′-end of the miRNA) to a complementary sequence in the 3′-UTR of its targeted mRNA (9). Sometimes additional pairing is needed at the 3′ of the miRNA to compensate non-Watson–Crick pairs called wobbles (14). Besides the complementarity and the conservation of the pairing sequences, some other factors may influence the pairing specificity and underlying function. For example, target sites surrounding long UTR edges were associated with lower expressed protein levels than those around the centre of the sequence (15). Besides, functional targets show a high proportion of adenines and uracils next to the binding site (16). Other basic factors highly related to active targets are miRNA cooperation (17), where a plausible effect in regulation is identified when several miRNAs are simultaneously bound to the same mRNA (rather than separately), and thermodynamic stability, where favourable energy is determined among the bound and unbound RNA double strand (18).

Several algorithms offer target prediction based on the combination of these conditions. They predict targets using miRNA and 3′-UTR sequences from selected protein coding transcripts known at that moment. The distinct approaches provide scores, energy or conservation values to highlight the reliability of the prediction. As each tool employs different criteria that govern a functional target, several integrative approaches emerged to offer these already calculated predictions combined, to ensure all possible restrictions. Some examples of these valuable efforts are MiRonTop (19), mirGator (20), mirWalk (21), MAGIA2 (22) or microRNA and mRNA Integrated Analysis (23). Many of them emphasize two of the most disturbing facts in the field, which are the lack of overlap between the different target prediction methods and the poor reliability found when predictions are validated using proteomics techniques.

The development of a tool based on a complete, consistent and unique dataset could avoid such problems increasing the reliability of miRNA and gene variants target studies (24). For this reason, we have developed miRGate, which uses a common dataset—rather than download pre-compiled data—to compute all possible targets from miRNAs sequences available in miRBase, and a complete 3′-UTR sequence dataset retrieved from EnsEMBL. Additionally, it also stores information of experimentally validated targets to test the reliability of predicted targets and provides valuable information to distinguish weak predictions.

To our knowledge, miRGate is the only available tool that addresses the little overlap among different targets using a common and an updated dataset. miRGate has been designed to jointly analyse miRNA and gene or gene variants lists in human, (including human viruses, such as Epstein–Barr and Kaposi sarcoma-associated herpes virus), mouse and rat to provide a novel catalogue of accurate in house predicted miRNA targets and programmatically access to the predictions in a massive way through RESTful web services.

## Methods

miRGate composed of diverse steps where data from different sources are processed and used as input for several algorithms. Results from these tools along with external information are converted and stored in a relational database. Scores from any individual prediction obtained from the different tools are processed to allow a comparison among algorithms results.

A schematic representation of all steps is shown in the Supplementary Figure S1.

### Sequence space

**Figure 1.**
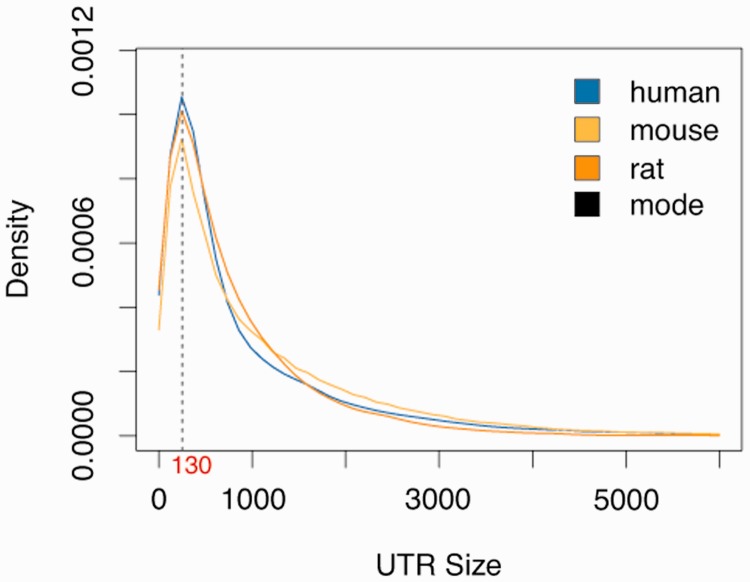
Distribution of known 3′-UTR sizes for human, mouse and rat. The statistical mode for human (142 bp), mouse (131 bp) and rat (122 bp). The average of these three values, which is ∼130 bp, was used from unknown 3′-UTRS.

To compute high reliable miRNA–mRNA targets, we created a consistent dataset of updated and complete sequences for miRNAs [based on miRBase 20 (25)] and 3′-UTR sequences for human, mouse and rat [based on EnsEMBL 74 (26)]. A complete summary of the 3′-UTR sequence dataset is presented in Table 1. Unlike other databases, we include in miRGate all known isoforms for all known genes stored in EnsEMBL, as each isoform can have an exclusive 3′-UTR. This contains, e.g. non-coding genes, pseudogenes [as they have been related to the regulation of the activity of cancer-related genes (27)] and mitochondrial RNAs, among others biotypes catalogued in Havana. A full comparison of sequences included in other databases/algorithms versus miRGate is presented in Supplementary Table S1. The untranslated sequences dataset used in this work are retrieved along with all provided annotations: HUGO Gene Nomenclature Committee name for human genes, gene and transcript names, genomic coordinates and Havana biotypes among others. Since not every transcript has a known UTR sequence, or some are smaller than 50 bp, 130 bp downstream from the end of the last exon were used as predicted UTR, as this size corresponds to the mode length of all known 3′-UTRs in human, mouse and rat (Figure 1). Additionally, miRGate provides protein structural information, functional and sequence conservation information for gene-oriented high throughput experiments using Annotating principal splice isoforms (28), which defines a principal variant: the gene isoform which is expressed in most of the tissues, for each gene in human, mouse and rat (29, 30).

**Table 1.** Total number of 3′-UTRs used in miRGate versus other databases/algorithms

Name	Build, year	Coding genes	Nc-genes	Pseudogenes	3′-UTR
miRanda	NCBI37, 2009	19 778	—	—	34 592
TargetScan	NCBI37, 2009	18 414	—	—	30 932
Pita	NCBI36, 2006	18 582	—	—	24 086
PicTar	NCBI35, 2005	20 254	—	—	20 254
miRGate	NCBI37, 2009	20 805	22 966	14 181	196 501

For miRNA sequences, we rely on miRBase 20 (25), which is the central database for miRNA sequence annotation and nomenclature registry. MiRBase 20 contains 24 521 pre-miRNAs, expressing 30 424 mature sequences in 206 species. In miRGate, we stored human, human viruses, mouse and rat miRNA sequences (Table 2), as well as other available information such as cleavage data from pre-miRNAs to mature miRNAs, genomic coordinates and family names.

**Table 2.** Total number of mature miRNAs included in the different datasets

Name	human	mouse	rat	Database Version
miRanda	1100	717	387	miRBase 15
TargetScan	1433	722	—	miRBase 17
Pita	692	500	—	miRBase 11
PicTar	81	81	81	Rfam 5
miRGate	2680	1983	763	miRBase 20

### Algorithms

One of our main motivations is to be able to determine accurate and novel targets from our own dataset. Although there are many freely available methods that provide miRNA target predictions for standard gene sequences, just a few of them allow prediction on provided sequences.

We compute miRNA target predictions using: (i) miRanda (31), which uses dynamic programming score alignments based on the complementary of nucleotides; (ii) Pita (32), which identifies full complementary seeds for each miRNA and calculates favourable energy among the bound and unbound double strand; (iii) RNAHybrid (33), that is based on favourable hybridization sites avoiding intramolecular duplexes; (iv) Microtar (34) that assess target sites based on RNA duplex energy calculation and (v) TargetScan (35), which scores predictions based on seed match, binding site localization and target conservation among the species. For Pita conservation score calculation, Phastcon hidden Markov model phylogenetic information (36) was added. In the case of TargetScan, EnsEMBL alignments for mammals were used (26). All information provided by the methods is stored, including target sites, energy scores, conservation scores, miRNA and mRNA coordinates and it is available for users. A complete description of the features included in each algorithm can be consulted in Table 3.

**Table 3.** Summary of the main features, scores and versions of the algorithms included in miRGate

Name	Type	Score	Version	Features
miRanda	Prediction tool	Energy > 140 kcal	3.3a	miRanda uses dynamic programming to score alignments based of the complementarity of nucleotides, allowing G-U wobble pairs.
Pita	Prediction tool	Conservation > 0.5	NA	Identifies initial full complementary seeds for each miRNA in the mRNA and computes the free energy of the unbound and bound double strand. It uses a phylogenetic hidden Markov model (34) called Phastcons; to filter out less conserved predicted target sites.
RNAHybrid	Prediction tool	Score > 0	2.2	Finds energetically most favourable hybridization sites avoiding intramolecular hybridization. Poisson approximation of multiple binding sites and calculation of effective numbers of orthologous targets in comparative studies of multiple organisms are assessed.
microtar	Prediction tool	Energy < 0 Kcal	NA	A program based on mRNA sequence complementarity and RNA duplex energy prediction by using Vienna package, assessing the impact of miRNA binding on complete mRNA molecules.
TargetScan	Prediction tool	Conservation in mammals	6	This algorithm requires perfect seed pairing to score the predictions according the type of the seed match, local AU contribution and mRNA binding site localization.
Tarbase	Validated target database	—	6	Contains detailed information for each miRNA–gene interaction, ranging from miRNA and gene-related facts to information specific to their interaction, including the experimental validation methodologies and their outcomes. All database entries are enriched with function-related data, as well as general information derived from external databases such as UniProt, Ensembl and RefSeq.
miRTarbase	Validated target database	—	4.5	It contains more than 51 000 validated miRNA-gene interactions which are collected by manually surveying pertinent literature retrieved by means of a text mining process aiming at research articles related to functional studies of miRNAs
miRecords	Validated target database	—	—	miRecords hosts a large, high-quality manually curated database of experimentally validated miRNA-target interactions with systematic documentation of experimental support for each interaction using text mining techniques.
OncomirDB	Validated target database	—	—	OncomirDB contains targets that have been validated and published in ∼9000 abstracts. A total number of 2259 manually curated entries with direct experimental evidences were stored.

### Experimentally validated data

**Figure 2.**
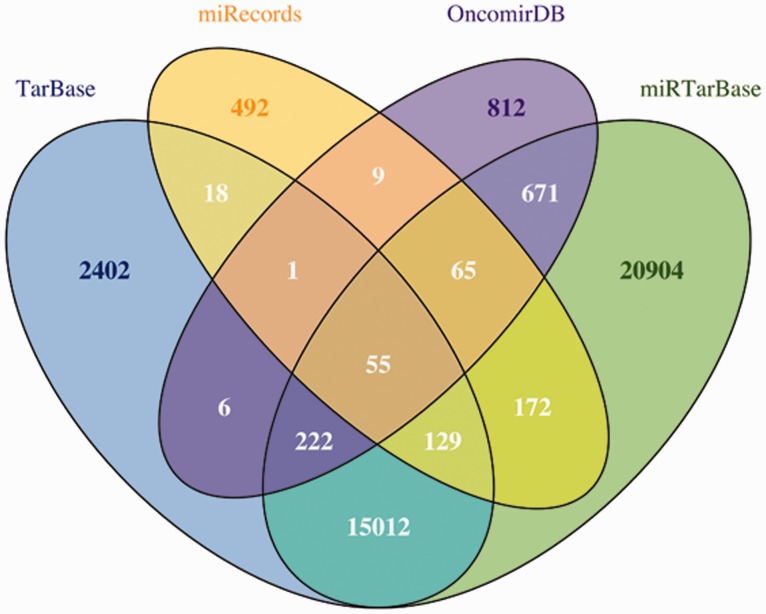
Venn diagram to represent the overlap between OncomirDB, Tarbase, miRTarBase and miRecords, four databases that compile experimentally validated miRNA–mRNA targets through article classification.

To contrast the predictions with experimentally validated miRNA–mRNA targets, miRGate also compiles information obtained with several validation methodologies and extracted from four different public databases: (i) Tarbase (37) and (ii) miRTarbase (38), which relay on text mining techniques to identify validated targets; (iii) miRecords (39), that manually curates targets mentioned in those publications selected using a systematic documentation strategy and (iv) OncomirDB (40), that publishes validated miRNA–mRNA targets by manually curating 9000 abstracts. In the case of human, the validated dataset from Tarbase (37), miRTarBase (38), miRecords (39) and OncomirDB (40) comprises 79 046 targets where only 40 991 (52%) of the mRNA–miRNA pairs are unique (Figure 2). A more detailed description of the experimental databases is shown in Table 3.

## Results

### Standardized prediction meta-score

The list of predictions (see Table 4 for a summary) is ranked by a *Z*-score that was computed by standardizing individual raw scores in each prediction among all predictions collected in the database. When more than one prediction algorithms in miRGate predict a identical target for the same miRNA and 3′-UTR in equivalent genomic coordinates, the results are combined generating a consensus weighted score (CWS) as it has been previously described (41).

$$\text{CWS}=\frac{\sum_{i}Z_{i}*W_{i}}{\sum_{i}W_{i}}$$

**Table 4.** Summary of the number of predictions organized by prediction tool and organism resulting of the execution by miRGate

	human	mouse	rat	
miRanda	34 838 559	16 164 311	1 372 897	
Pita	773 112	313 113	52 281	
RNAHybrid	36 832 689	10 390 354	536 248	
microtar	6 049 837	1 750 058	3 348 100	
Targetscan	7 270 936	5 186 036	417 501	
TarBase	36 853	20 513	7	
miRTarbase	39 118	9 314	307	
miRecords	1 198	227	—	
OncomirDB	2 368	1 917	—	
miRGate	85 844 670	33 835 843	5 727 341	125 407 854

For each identical prediction, obtained for a different algorithm, let *Z*_i_ be the standardized score produced by that tool and *W*_i_ corresponds to the probability that an above-the-score prediction is not a false positive, given the complementary cumulative distribution of scores shown by the *i*th tool when comparing its predictions against a dataset of validated targets.

This approach was found to improve the reliability of predictions from different methods that although different in nature, reflects in this particular case, the probability of a miRNA to bind to a complementary sequence of an mRNA region.

### Validation

**Figure 3.**
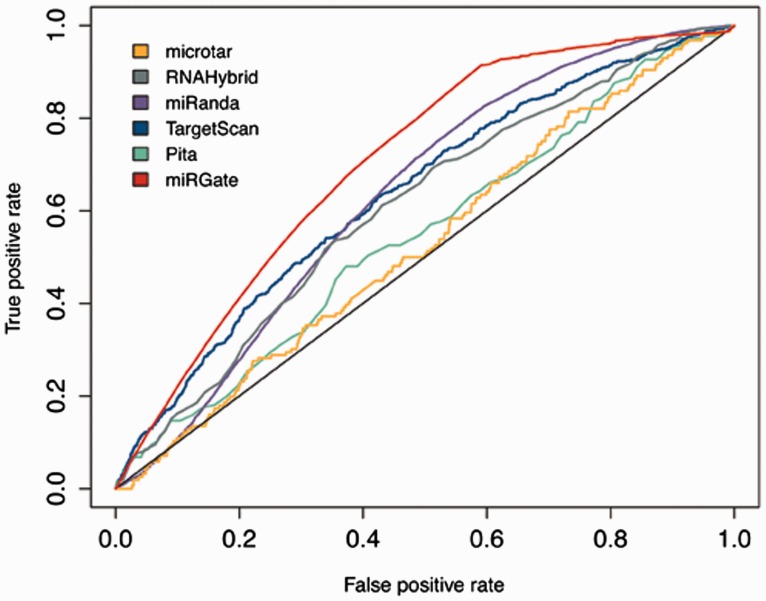
ROC curve illustrating the performance of miRGate and each individual method separately, over four datasets of validated targets: OncomirDB, miRecords, Tarbase and miRTarBase. The AUC obtained for each method is: microtar: 0.528, RNAHybrid: 0.609, miRanda: 0.632, TargetScan: 0.638, Pita: 0.548 and miRGate: 0.704.

**Figure 4.**
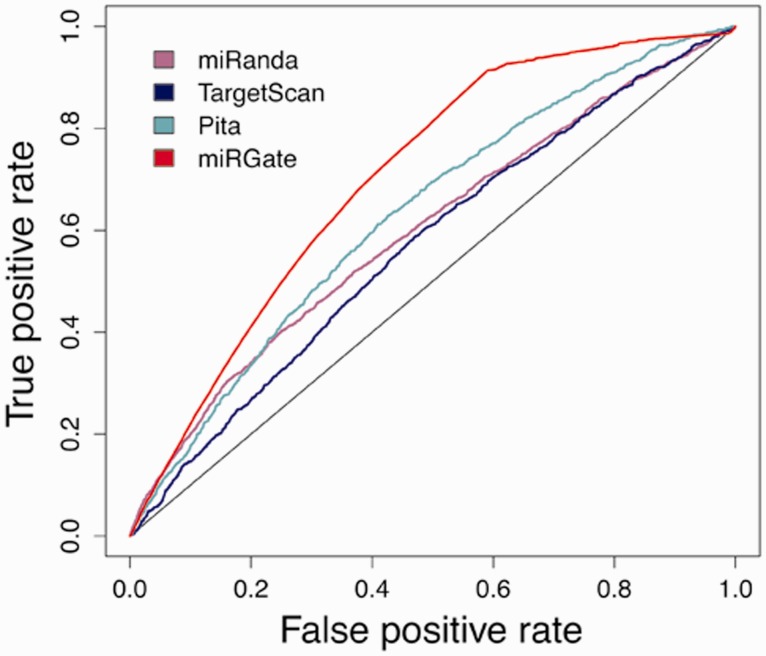
Integration of miRGate predictions versus downloadable predictions from each individual method (only available for miRanda, Targetscan and Pita) over validated targets. The best resulting datasets where selected for each method: miRanda (purple): good scores and conserved targets (AUC: 0.599). Targetscan (blue): conserved targets (AUC: 0.560) and Pita (light green): top scores (AUC: 0.630). miRGate (red, AUC: 0.704).

Although miRGate uses established and well-known prediction algorithms, we evaluated the predictions obtained by those methods against a dataset of experimentally validated targets. *Z*-scores and consensus-weighted scores were plotted using ROC (receiver operating characteristic) (42). The integrative approach designed in miRGate outperforms the result of each method separately (Figure 3). Outperformance increases more drastically when miRGate predictions are then compared against available pre-compiled targets, obtaining an average increment of 10%. The true-positive rate is even better, when the false positive rate is over 0.6. (Figure 4).

**Figure 5.**
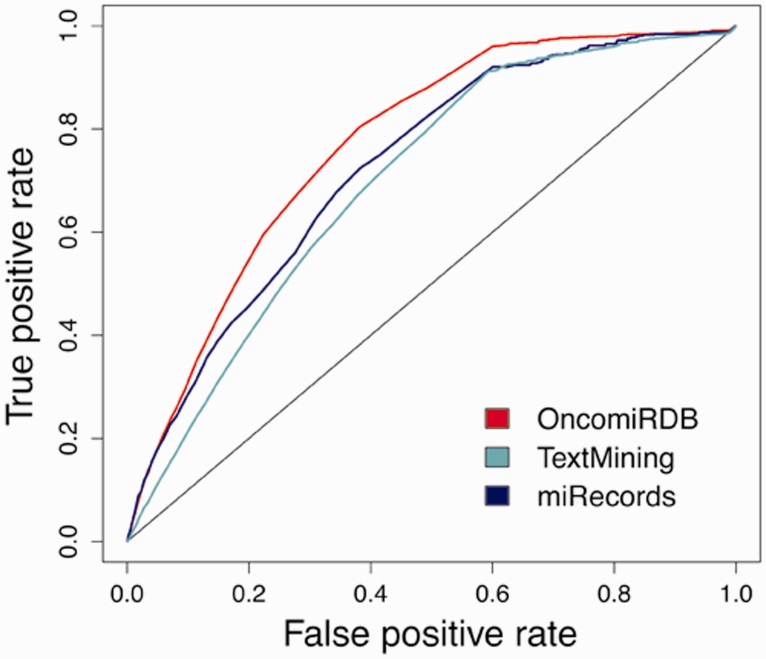
Accuracy achieved when validated databases are distributed according to a reliable criterion. OncomirDB, AUC of 0.769, based on manually curation (high reliability), miRecords, AUC of 0.727, as a partially curated database (medium reliability) and miRTarBase and Tarbase, AUC of 0.699, relying on text mining techniques (lower reliability).

We also observed that better accuracy is obtained when target prediction results are contrasted with the more confident targets. In that sense, datasets were divided according to a reliability criteria: (i) OncomirDB (40) as a manually curated database (highly reliable), (ii) miRecords (39) as a partially curated dataset (medium reliability) and (iii) a combined dataset comprised two text mining prediction sources, mirTarbase (38) and Tarbase (37), as low reliability. The area under the curve (AUC) rises from 0.6, in low reliable, to 0.78 in high confident targets (Figure 5).

In summary, the incorporation of this complete dataset in miRGate has improved the prediction reach of the individual methods (a 10–21% improvement in performance), as seen by the comparison of the whole set versus individual methods when using experimental confirmed datasets. This improvement is even notorious when we compared the data in our database against the pre-compiled datasets that other integrative methods employ.

Moreover, miRGate has been successfully applied to independent datasets providing predictions that were validated using different experimental techniques from diverse transcriptome profiling technologies (such as microarrays, RNA-Seq or miRNA-Seq). To date, eight different works have successfully validated miRGate targets using different experimental procedures (43–50).

### Web interface

miRGate database can be accessed through a web page to search for potential targets to their genes and/or miRNAs of interest.

The page is designed as an intuitive step-by-step form where users fill basic information such as organism and gene/miRNA names using gene symbols, miRNAs names, miRNAs accessions, EnsEMBL genes, EnsEMBL transcript Identificators or even probe names from different expression array platforms. To unify entity nomenclature and make easier the data introduction, the web page includes a type-ahead function that allows selecting miRNAs or genes names included in miRGate, similar to the provided input. As an optional step, miRGate provides an advanced feature where several filtering options can be adjusted. Among them, we highlight the possibility to filter by ENCODE principal isoforms (29), HAVANA biotypes and/or predicted 3′-UTR mRNA sequences. We also provide a novel feature, not present in other methods, that considers an overlap when the binding event between the miRNA seed and the mRNA 3′-UTR occurs in the same genomic position. Hence, it is possible to label remarkably agreed predictions when two or more different algorithms coincide predicting the same target in terms of target site type and RNA coordinates.

It is worth mentioning that those predictions that have been found to be experimentally corroborated (i.e. contained in at least one of the four experimental databases incorporated in miRGate) are highlighted in bold in the web page to make their identification easier to the user. Besides, for each 3′-UTR, we provide links to APADB (51), a database for alternate polyadenylation that provides information of potential loss of miRNA binding sites.

All results can be saved in csv format for downstream analyses. Details regarding the number of miRNAs and 3′-UTRs in comparison with other integrative analysis are provided in Supplementary Table S1.

### RESTful API

Representational state transfer (REST) is often used as an alternative to Simple Object Access Protocol to deploy web services (52). miRGate provides a EXtensible Markup Language-based REST application programming interface (API) to allow automated queries in the database using remote programmatic tools. Using this interface, the server can be accessed from multiple programming languages, allowing researchers to wire miRGate results to their analysis pipelines. The current API version allows gene/miRNAs retrieval operations (as cleavage information, gene localization or seed sequence recovering for miRNAs or isoform localization, ENCODE annotation or Havana biotype for genes), including data sources listing, catalogue listing and query execution to retrieve detailed information about predicted and validated targets sites.

Details and examples of the implementation of the RESTful miRGate API in the Perl language are provided in the online documentation (http://mirgate.bioinfo.cnio.es/API/api.html).

## Discussion

The aim of miRGate is to provide a reliable miRNA–mRNA pairs database and at the same time to fill the gap among predicted and non-concordant experimentally validated targets. At present, existing alternatives rely on pre-compiled targets from external resources. As an example, mirGator (20) uses a human dataset with pre-compiled targets from Pita (32), PicTar (53), TargetScan (35) and miRanda (31), which implies three different human builds and hence a different and a dissonant number of 3′-UTR sequences. mirWalk (21) calculates possible targets using RNAHybrid (33) software, but as other databases, it combines the results with previous computed targets from different sources and consequently discordant datasets. Since a considerably increase of overlap is obtained among target predictions or validated pairs lists when prediction methods are run using a common source of annotation (24), we designed miRGate database to use a complete dataset built on up-to-date sources that provide full miRNA and 3′-UTR sequences. Our dataset was used as a common input for five different public algorithms that predict miRNA–mRNA targets and integrated in a relational database. To our knowledge, miRGate is the only available tool that reconciles the existing disagreement among predicted pairs and experimental validated pairs. The methodology implemented in miRGate, resulted in an increase of 10–21% in accuracy when our predictions are compared to pre-compiled datasets employed by other tools versus a dataset of validated miRNA–mRNA targets.

It is also important to note that miRGate database, unlike other tools, includes all variants of every gene in human, mouse and rat that potentially could be expressed in any experimental condition (including pseudogenes, antisense transcripts, non-coding genes among others). Others focus on protein coding isoforms or the longest protein-coding variant, underrating the number of regulatory elements of the gene. A complete 3′-UTR dataset is essential as these regions contain several regulation motifs that control the expression and harbour miRNA binding sites and/or other regulatory sequences. Longer 3′-UTRs will more likely possess such signals, or more of them, and the mRNA will likely be more subjected to regulation (54). Furthermore, the length of the 3′-UTR can affect not only the stability but also the localization, transport and translational properties of the mRNA (55). Other important reason that supports a complete dataset inclusion is based on the restriction rules that dictate an effective target site; for instance, binding positions over the 3′-UTR, AU enrichment and miRNA binding cooperation along the 3′-UTR sequence. As these features are sequence dependent and a gene may have several and different 3′-untranslated sequences, the real regulation by miRNAs should be determined taking into account all 3′-regulatory sequences. Poliseno *et al*. (26) confirmed this observation, where a pseudogene was found to be responsible of a miss-regulation of *PTEN1*. For this reason, the inclusion in miRGate of all variants allows us to provide a complete and undistorted regulation network that potentially controls cellular processes where gene isoforms are expressed.

miRGate includes miRNAs virus–host target gene pair’s prediction such as Epstein–Barr and Kaposi sarcoma-associated herpesvirus. Little information is found about these viruses as most of other databases focus on intra-organism target predictions, but miRGate calculated pairs were successfully validated in diffuse large B-cell lymphomas (42) and Burkitt lymphoma samples infected with Epstein–Barr virus miRNAs (43). Apart from viruses, miRGate has also been used in hereditary breast tumour samples, hyperdiploid multiple myelomas, mantel cell lymphomas and B-cell lymphomas where expression levels of isoforms and/or miRNAs were measured using distinct techniques. In all cases, miRGate provided targets that were confirmed, pointing the suitability of this tool to the scientific community (43–50).

In addition, miRGate can be accessed as a RESTful API, enabling the integration and inter-operation of diverse sources based on related technology. miRGate API is designed to provide all stored information and it can be implemented with other catalogued services in analyses pipelines. We believe that this could be a very helpful tool as it offers a fast, automatic, customizable and integrated query execution.

To summarize, miRGate is a unique catalogue of reliable in-house-predicted miRNA targets and also experimentally validated pairs for the scientific community that is publicly available, either as a web page or as a RESTful web service. It includes a common, complete and updated dataset from miRNAs and all known gene variants for human, mouse and rat providing high confident predictions. Of note, miRGate succeed to provide useful targets obtained from different transcriptomic techniques that were robustly validated.

## Supplementary Data

Supplementary data are available at *Database* Online.

Supplementary Data
