# Supplementary material for: miRGate: a curated database of human, mouse and rat miRNA–mRNA targets
Source: Database (Oxford). 2015 Apr 8;2015:bav035. doi: 10.1093/database/bav035 (PMC4390609; doi:10.1093/database/bav035)
Supplement: Supplementary Data [file supp_2015_bav035_index.html]

miRGate: a curated database of human, mouse and rat miRNA–mRNA targets — Supplementary Data 

# miRGate: a curated database of human, mouse and rat miRNA–mRNA targets

## Supplementary Data

files

**Files in this Data Supplement:**

- Supplementary Data - doc file
- Supplementary Data - doc file
