# Supplementary material for: miRGate: a curated database of human, mouse and rat miRNA–mRNA targets
Source: Database (Oxford). 2015 Apr 8;2015:bav035. doi: 10.1093/database/bav035 (PMC4390609; doi:10.1093/database/bav035)
Supplement: Supplementary Data [file supp_bav035_Supplementary_Figure_1.doc]

**Supplementary Figure 1.**


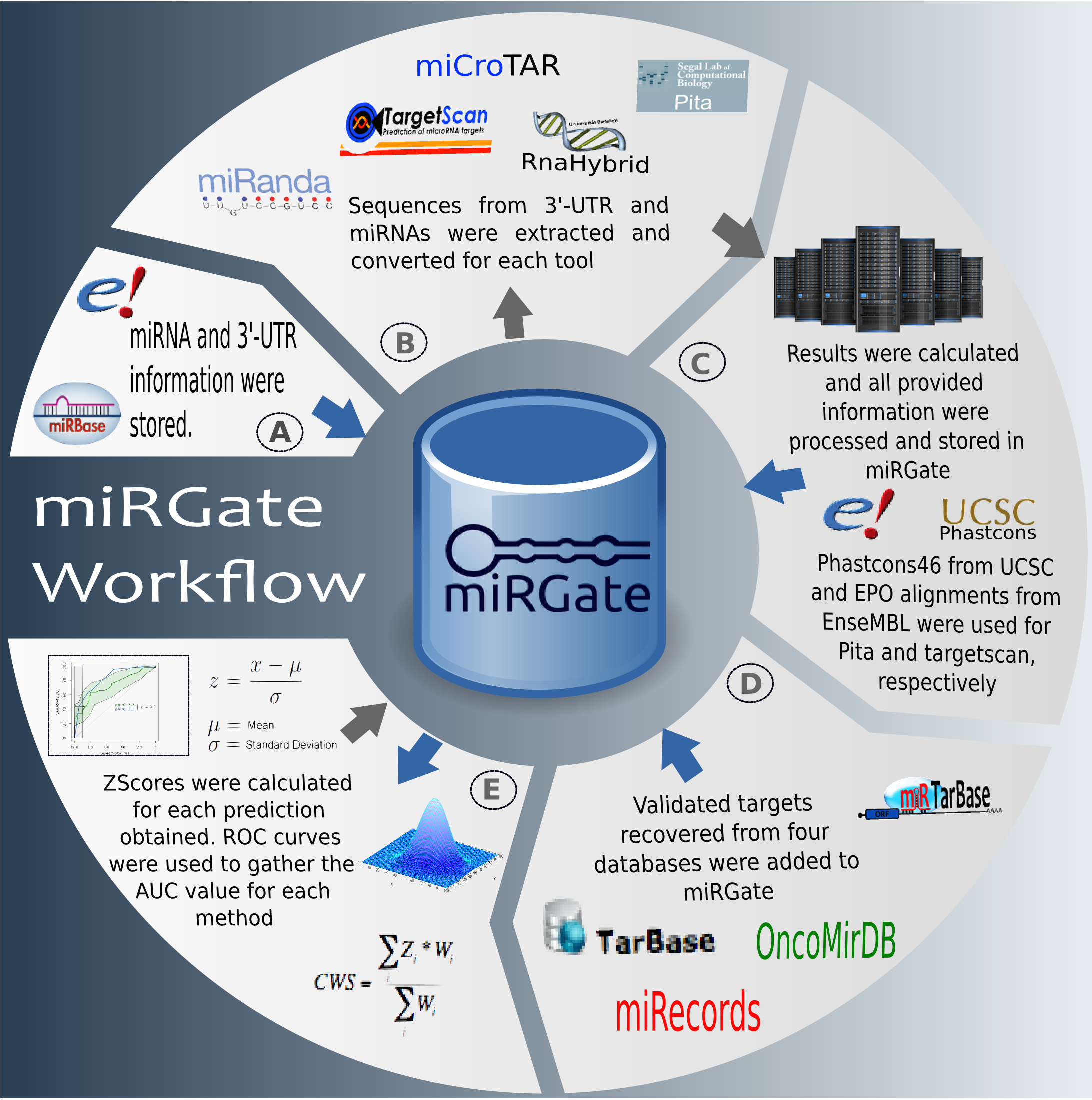
**Schematic representation of miRGate workflow**. (A) Sequence dataset is composed of all gene information from EnsEMBL 74 and all miRNA data from miRBase 20. This information is stored in the miRGate database. (B) Stored information (sequences and additional data) is processed from the database and converted into the specific input files of each algorithm (miRanda, RNAHybrid, Pita, microtar and Targescan). (C) These algorithms are executed in a high performance computer. Additional needed information as Phastcons conservation scores in the case of Pita or EnsEMBL EPO (Enredo, Pecan/Ortheus) alignments in the case of Targetscan are also added to the calculations. The obtained results from the different algorithms are processed and inserted in the database along with all provided information by these tools (scores, energy, 3’UTR coordinates, etc). (D) External validated targets from miRecords, OncomirDB, Tarbase and miRTarBase are included in the database. (E) Scores from each calculated predictions are processed to obtain z-scores. Predicted targets and their z-scores are examined using ROC curves against the validated dataset. The area under the curve (AUC) is used to calculate a consensus weighted score (CWS) for those coinciding predictions obtained for more than one tool in the same genomic positions.
