# Supplementary material for: miRGate: a curated database of human, mouse and rat miRNA–mRNA targets
Source: Database (Oxford). 2015 Apr 8;2015:bav035. doi: 10.1093/database/bav035 (PMC4390609; doi:10.1093/database/bav035)
Supplement: Supplementary Data [file supp_bav035_Supplementary_Table_1.doc]

| **Database Name** | | **Publication Year** | **Number of methods (Dataset year)** | | | | **Number of validates DB's** | | **Number of Coding Genes** | | **Number of Pseudogenes** | | **Number of Non Coding Genes** | | | **Total number of UTR's** | | | **Isoforms** | | | **ENCODE principal Isoforms** | | | **Havana Biotypes** | | | | | **miRBase version** | | | **In-house calculated predictions** | | |
| --- | --- | --- | --- | --- | --- | --- | --- | --- | --- | --- | --- | --- | --- | --- | --- | --- | --- | --- | --- | --- | --- | --- | --- | --- | --- | --- | --- | --- | --- | --- | --- | --- | --- | --- | --- |
| miRGator | | 2013 | 6 | 2004/2010 | | 3 | | 21370 | | | | 🗷 | 🗷 | | | 21370 | | | 🗷 | | | 🗷 | | | 🗷 | | | | - | | | | 🗷 | | |
| Magia2 | | 2012 | 8 | 2006/2011 | | 3 | | 20930 | | | | 🗷 | 🗷 | | | 20930 | | | 🗷 | | | 🗷 | | | 🗷 | | | | 18 | | | | 🗷 | | |
| microPIR | | 2012 | 1 | 2006 | | 0 | | 22360 | | | | 🗷 | 🗷 | | | 22360 | | | 🗷 | | | 🗷 | | | 🗷 | | | | 13 | | | | 🗹 | | |
| mirConnX | | 2011 | 5 | 2006 | | 2 | | 21370 | | | | 🗷 | 🗷 | | | 21370 | | | 🗷 | | | 🗷 | | | 🗷 | | | | 14 | | | | * | | |
| miRTar | | 2011 | 4 | - | | 0 | | 32123 | | | | 🗷 | 🗷 | | | 32123 | | | 🗷 | | | 🗷 | | | 🗷 | | | | 15 | | | | 🗹 | | |
| miTALOS | | 2011 | 5 | 2007/2010 | | 0 | | 21370 | | | | 🗷 | 🗷 | | | 21370 | | | 🗷 | | | 🗷 | | | 🗷 | | | | - | | | | 🗷 | | |
| miRWalk | | 2011 | 8 | 2007/2010 | | 1 | | 21370 | | | | 🗷 | 🗷 | | | 21370 | | | 🗷 | | | 🗷 | | | 🗷 | | | | - | | | | 🗷 | | |
| RepTar | | 2010 | 1 | 2010 | | 0 | | 18421 | | | | 🗷 | 🗷 | | | 18421 | | | 🗷 | | | 🗷 | | | 🗷 | | | | 15 | | | | 🗹 | | |
| miRGate | | - | 5 | 2014 | | 4 | | 20805 | | | | 14181 | 22966 | | | 196501 | | | 🗹 | | | 🗹 | | | | 🗹 | | | 20 | | | | | 🗹 | |
|  | |  |  | |  | |  | | |  | | |  |  | | |  | | |  | | |  | | | |  | | | |  | | | |  |
|  | * The target is predicted if the input UTR is not in the predicted database. | | | | | | | | | | | |  | |  | | |  | | |  | | |  | | | |  | | | |  |  | | |
